# Genetic diversity, population structure and historical demography of the two-spined yellowtail stargazer (*Uranoscopus cognatus*)

**DOI:** 10.1038/s41598-021-92905-6

**Published:** 2021-06-25

**Authors:** Nur Ilham Syahadah Mohd Yusoff, Tun Nurul Aimi Mat Jaafar, Veera Vilasri, Siti Azizah Mohd Nor, Ying Giat Seah, Ahasan Habib, Li Lian Wong, Muhd Danish-Daniel, Yeong Yik Sung, Abd. Ghaffar Mazlan, Rumeaida Mat Piah, Shahrol Idham Ismail, Min Pau Tan

**Affiliations:** 1 Faculty of Fisheries and Food Science, Universiti Malaysia Terengganu, 21030 Kuala Nerus, Terengganu Malaysia; 2 Natural History Museum, National Science Museum (Thailand) Technopolis, Khlong 5, Khlong Luang, 12120 Pathumthani Thailand; 3 Institute Marine Biotechnology IMB, Universiti Malaysia Terengganu, 21030 Kuala Nerus, Terengganu Malaysia; 4 South China Sea Repository and Reference Centre, Institute Oceanography and Environment, Universiti Malaysia Terengganu, 21030 Kuala Nerus, Terengganu Malaysia; 5 Department of Fisheries and Marine Science, Noakhali Science and Technology University, Noakhali, 3814 Bangladesh; 6 Faculty of Science and Marine Environment, Universiti Malaysia Terengganu, 21030 Kuala Nerus, Terengganu Malaysia

**Keywords:** Ecology, Genetics, Zoology

## Abstract

Benthic species, though ecologically important, are vulnerable to genetic loss and population size reduction due to impacts from fishing trawls. An assessment of genetic diversity and population structure is therefore needed to assist in a resource management program. To address this issue, the two-spined yellowtail stargazer (*Uranoscopus cognatus*) was collected within selected locations in the Indo-West Pacific (IWP). The partial mitochondrial DNA cytochrome c oxidase subunit 1 and the nuclear DNA recombination activating gene 1 were sequenced. Genetic diversity analyses revealed that the populations were moderately to highly diversified (haplotype diversity, H = 0.490–0.900, nucleotide diversity, π = 0.0010–0.0034) except sampling station (ST) 1 and 14. The low diversity level, however was apparent only in the matrilineal marker (H = 0.118–0.216; π = 0.0004–0.0008), possibly due to stochastic factors or anthropogenic stressors. Population structure analyses revealed a retention of ancestral polymorphism that was likely due to incomplete lineage sorting in *U. cognatus*, and prolonged vicariance by the Indo-Pacific Barrier has partitioned them into separate stock units. Population segregation was also shown by the phenotypic divergence in allopatric populations, regarding the premaxillary protrusion, which is possibly associated with the mechanism for upper jaw movement in biomechanical feeding approaches. The moderate genetic diversity estimated for each region, in addition to past population expansion events, indicated that *U. cognatus* within the IWP was still healthy and abundant (except in ST1 and 14), and two stock units were identified to be subjected to a specific resource management program.

## Introduction

Fishing is one of the most significant causes of decline in populations of ocean species. Catching fish is not harmful to the ocean, except for when we catch so many fish that there are not enough to breed and replenish themselves, including incidental removal of non-target fish species or younger fish. The damage from overfishing and bycatch affects not only the marine environment and socio-economy of the fishermen and their communities, but also billions of people who rely on fish for protein^1^. In Malaysia, a large amount of bycatch is continuously landed each year, representing the biggest component of the total marine fish landing. Fisheries statistics for 2010–2019 indicate a total of 1,464,917.3 tons (average) of fish landings in Malaysia, of which, 18.4% comprised non-target species^2^. Of these, 80.1% of the bycatch was landed by trawl nets, followed by purse seines and other seines, suggesting the harmful effect of fishing trawls on fishery resources. Meanwhile, fisheries statistics on the west coast of Thailand (WCT) revealed a slightly higher relative proportion of the bycatch for the same period of the year, where on average, 22.2% of the total marine fish landing (an average of 299,032 tons) comprised non-target species^3^.

**Figure 1:**
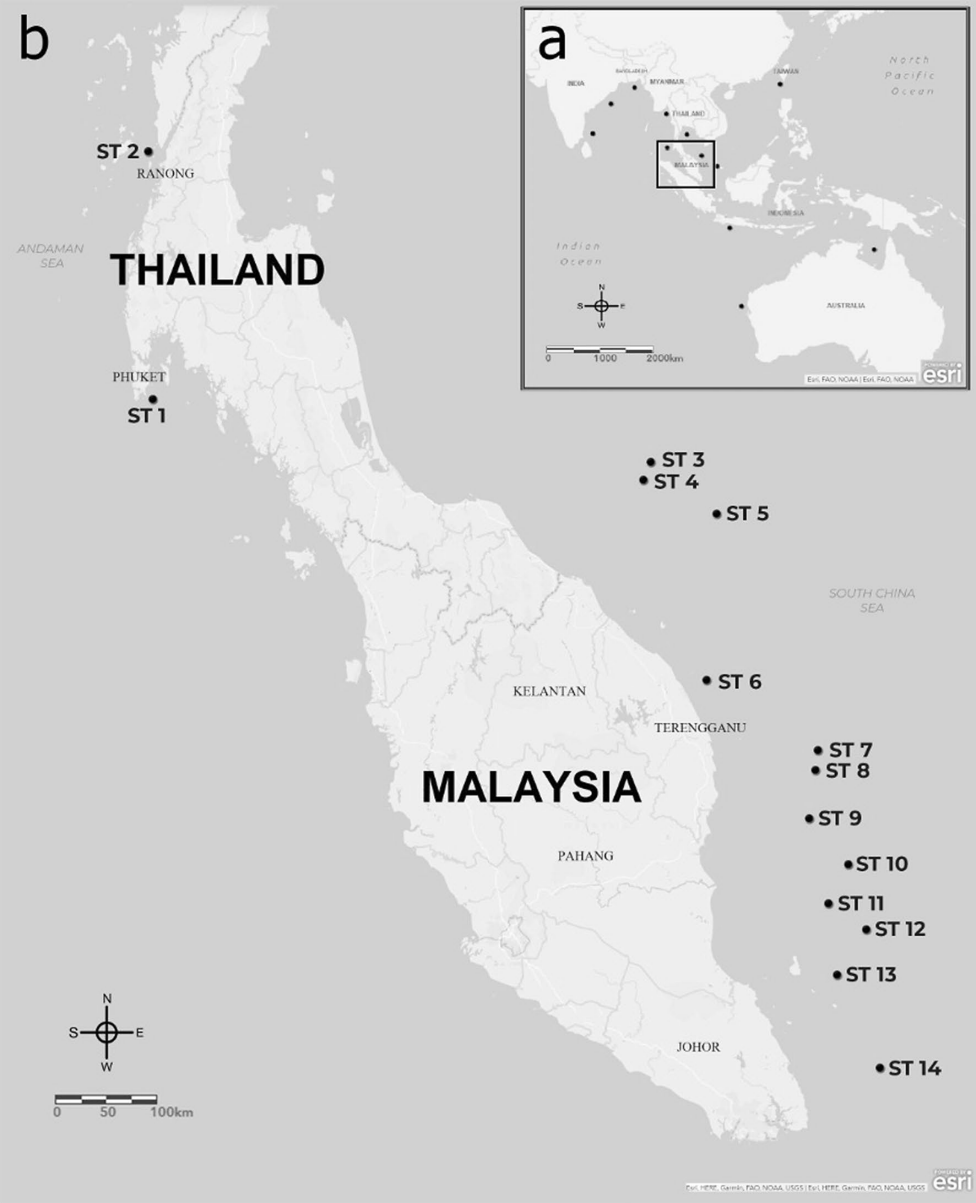
Distribution of *Uranoscopus cognatus* (**a**) based on reports of occurrence (indicated by black dots). Squared area indicates sampling locations in this study (**b**) Fourteen sampling stations (ST) of *U. cognatus* divided into two geographical regions i.e. the west coast of Thailand (WCT) (ST1-2) and the east coast of Peninsular Malaysia (ECPM) (ST3-14). (The map was created in ArcGIS Online, https://www.esri.com/en-us/arcgis/products/arcgis-online/resources).

Benthic species, though ecologically important, are often a major component of the bycatch of fishing vessels^4–6^. Uncontrolled or incidental bycatch activities may kill large numbers of unintended species, leading to a serious threat to marine diversity as a whole^5^. This includes benthic dwelling marine fish, such as the two-spined yellowtail stargazer (*Uranoscopus cognatus*). Assessment of its populations in the central-eastern coast of India revealed a high exploitation ratio due to the high discard rates in the bycatch and overfishing^4^. A high exploitation rate due to overfishing was also reported in the Atlantic stargazer (*U. scaber*) in the southeastern Black Sea region^7^. Continuous fishing pressure may ultimately lead these marine species to a high risk of genetic and population bottlenecks, if preventive action is not taken^4^. *U. cognatus* is commonly landed as bycatch and discarded in huge quantities in Malaysia and Thailand, as it has a low economic value. Nevertheless, to date, information on its populations particularly from this geographical region, is unavailable. Specifically, there is no available data on the population genetics of this species, despite its vast distribution areas. This species was reported to occur in the eastern Indian Ocean and the west Pacific Ocean from India, east to Northern Australia, south to the Philippines and north to Taiwan^4,8,9^ (Fig. 1a). Thus, there is a need to establish baseline data to understand its current stock status.

Stargazers (Uranoscopidae: Perciformes) are characterized by a heavily armored and flattened head with dorsally directed eyes (hence, stargazing)^10,11^. They are capable of burying into sandy or muddy substrates and use specialized appendages (worm-shaped lures) fishing gear to attract its usual prey of small fishes and crustaceans^12–14^. This family comprises seven genera, of which two are monotypic and distributed worldwide in tropical and temperate oceans^15,16^. Currently, there are 55 validated species in the family^17^. The genus *Uranoscopus* Linnaeus, 1758 is the most specious group of stargazers, consisting of 25 described living species^17,18^, with the highest number of species found in the Indian Ocean^19^. Of these, four species have been recorded in the surrounding seas of Malaysia^20^, namely *U. bicinctus* Temminck and Schlegel, 1843, *U. cadenati* Poll, 1959, *U. cognatus* Cantor, 1849 and *U. oligolepis* Bleeker, 1878. Species identification within this genus is very challenging due to the highly overlapping morphological characteristics among species, except for *U. cognatus*, which has two unique and distinguishing pairs of basipterygial processes with sharp spurs and a short black cirrus on the eyes^12,15^. *U. cognatus* is one of the smallest and most slender species within the genus.

Biogeographically, the Andaman Sea (marginal sea of the Indian Ocean) and South China Sea (marginal sea of the West Pacific Ocean) are situated within the Indo-Pacific (sometimes known as Indo-West Pacific (IWP)) where the Southeast Asian region lies^21^. During the Pleistocene glacial cycles, a series of elevations and depressions in the sea level as low as 120 m below the present level, exposed the Sundaic region (extension of the Southeast Asian region), resulting in the emergence of land barriers that isolated the South China Sea from the Indian Ocean at its southern limit and from the Sulu Sea to the east^22–24^. Consequently, the terrestrial, freshwater, and marine taxa were affected by this historical geomorphological rearrangement, shaping the genetic distribution that is apparent today. Several hypotheses on molecular biogeography of multispecies within the IWP have been proposed^25^; of these, several studies advocate genetic homogeneity or partial panmixia restricted within a particular area of the ocean, for example in moray eels^26^, Indian mackerel (*Rastrelliger kanagurta*)^27^ and Japanese scad (*Decapterus maruadsi*)^28^, while others indicate extensive structuring, including in reef-fishes^29–32^, invertebrates^33–35^, seahorses^36^ and pelagic fish^37^.

Population genetic tools offer an opportunity to elucidate the genetic level, patterns of dispersal (connectivity), and demographics (past and present), to better understand species responses’ to ecological changes and anthropogenic stressors^38,39^. Thus, the multi-locus genetic approach is increasingly favored due to the high success rate in revealing both the contemporary and historical events for targeted species^26,29,33,40–46^. Specifically, mitochondrial DNA (mtDNA) markers are maternally inherited and are thus characterized by a low rate of recombination^47^, relatively rapid rate of evolution, and a deeper and greater range in a bifurcating evolutionary tree^48,49^. However, nuclear DNA (nuDNA) markers have a lower substitution rate^50^, are highly conserved^51^ and have a larger effective population size compared to mtDNA^52^. Due to these different characteristics, gene genealogies obtained from individual DNA marker can be different from the actual evolutionary history of a species, thus inferring that both paternal and maternal DNA markers are essential in providing a better understanding of evolutionary history^48^. The morphological approach is one of the simplest, most cost-effective and most efficient methods to identify fish stock structure and discriminate species, which is determined using differences in body measurements, meristic and anatomical characteristics^53,54^. Both genetic and phenotypic-based methods are imperative in designing sound management strategies, thus setting priorities for rational exploitation^21,22^.

To date, only a single genetic study describing the whole mitogenome of *U. cognatus* is available^55^, limiting our knowledge on the population genetic diversity of this bioresource. In the study, two samples each from the west coast of Thailand and east coast of Peninsular Malaysia were analyzed. The final partitioned nucleotide alignment consists of 14,098 base pairs, and formed a maximally-supported monophyletic group within the *Uranoscopus* clade, although clustering between samples collected from Malaysia was not strongly supported^55^. Herein, the mtDNA CO1 and nuDNA RAG1 genes were sequenced to examine the genetic diversity, phylogenetics and demography of *U. cognatus* within the IWP, with the inclusion of CO1 sequences retrieved from Indonesia (INDO) and Australia (AUS). A quantitative morphological study was also conducted to investigate whether allopatric *U. cognatus* differ morphologically. The hypotheses for this study were that a low genetic diversity would be expected due to the high exploitation rate, and a genetic homogeneity for intra-regional populations would be observed due to the absence of a physical barrier, yet a significant population structure between inter-regional populations would be expected. The data obtained from this study will be beneficial for establishing baseline data for stock management.

## Results

### Genetic diversity

The mtDNA CO1 dataset revealed a total of 38 polymorphic sites (12 parsimoniously informative and 26 singletons) defining 43 putative haplotypes, of which 33 (76.7%) were unique sequences. Point mutations occurred at the first and third codon positions, resulting in six non-synonymous amino acid substitutions. The number of haplotypes (nh) ranged from two (sampling station (ST)1) to 13 (ST7) (Table 1), with an average of three haplotypes per ST. The haplotypes were regionally specific, *i.e.* Hap29–32 were private to the west coast of Thailand (WCT), and the rest were present only on the east coast of Peninsular Malaysia (ECPM). Hap01 (59.13%) and Hap29 (86.96%) were the most common haplotypes in the ECPM and WCT. The nuDNA RAG1 sequence data revealed 22 polymorphic sites (11 parsimoniously informative and 11 singletons), defining 33 putative haplotypes, of which 21 (63.6%) were singletons. Nucleotide substitutions occurred at all codon positions, resulting in 14 non-synonymous amino acid substitutions. The number of haplotypes ranged from three at ST2 to nine at ST3, 9 and 11 (Table 1). All five RAG1 haplotypes that were present in the WCT (Hap05, 07, 08, 10 and 17) were also found in the ECPM.

**Table 1:** Genetic polymorphisms and neutrality tests of *Uranoscopus cognatus* inferred from the mitochondrial DNA CO1 (534 base pairs) and nuclear DNA RAG1 (1,426 base pairs) sequences.

Region	ST	mtDNA CO1							nuDNA RAG1							
		N	Genetic polymorphism				Neutrality test		N	Genetic polymorphism					Neutrality test	
			S	nh	H	π	Tajima’s *D*	Fu’s *Fs*		S		nh	H	π	Tajima’s *D*	Fu’s *Fs*
WCT	1	17	2	2	0.118	0.0004	− 1.504	0.122	17	3		5	0.728	0.0008	0.7819	− 0.023
	2	6	2	3	0.600	0.0013	− 1.132	− 0.858	6	2		3	0.733	0.0008	1.393	0.0203
	***Total***	23	4	4	0.249	0.0007	− 1.881*	− 2.270	23	3		5	0.723	0.0008	0.996	− 0.622
ECPM	3	17	3	4	0.493	0.0010	− 1.096	− 1.537*	17	9		9	0.860	0.0013	− 1.155	− 4.374*
	4	18	6	6	0.490	0.0014	− 1.849*	− 3.313*	18	7		8	0.699	0.0011	− 0.778	− 3.408*
	5	18	8	7	0.634	0.0022	− 1.766*	− 3.296*	11	4		4	0.673	0.0009	− 0.249	− 0.228
	6	9	5	4	0.583	0.0021	− 1.677*	− 0.822	N/A	N/A		N/A	N/A	N/A	N/A	N/A
	7	20	12	13	0.884	0.0032	− 1.791*	− 11.021*	5	3		4	0.900	0.0008	− 1.049	− 1.938*
	8	18	7	8	0.797	0.0024	− 1.235	− 4.203*	17	5		7	0.772	0.0009	− 0.532	− 3.160*
	9	19	3	4	0.573	0.0013	− 0.459	− 0.823	19	7		9	0.731	0.0011	− 0.843	− 4.815*
	10	18	7	6	0.562	0.0016	− 1.933*	− 2.867*	5	4		4	0.900	0.0013	− 0.410	− 1.195
	11	18	11	9	0.804	0.0034	− 1.576	− 4.121*	17	14		9	0.868	0.0018	− 1.461	− 2.906
	12	19	6	6	0.643	0.0017	− 1.583*	− 2.763	11	5		7	0.818	0.0011	− 0.404	− 3.787*
	13	16	4	5	0.600	0.0013	− 1.312	− 2.363*	14	4		5	0.593	0.0006	− 0.905	− 1.870*
	14	18	4	3	0.216	0.0008	− 1.853*	− 0.507	14	6		7	0.692	0.0009	− 1.081	− 3.466*
	***Total***	208	37	39	0.628	0.0019	− 2.418*	− 57.825*	148	22		33	0.749	0.0011	− 1.757	− 33.950*
**Overall**		231	38	43	0.692	0.0024	− 2.278*	− 58.217*	171	22		33	0.789	0.0011	− 1.645	− 33.320*

*Significant at P < 0.05.

*ST* sampling station, *N* sample size, *S* number of segregating site, *nh* number of haplotype, *H* haplotype diversity, *π* nucleotide diversity, *WCT* west coast Thailand, *ECPM* east coast Peninsular Malaysia, *N/A* data not available.

Both genetic markers revealed that *U. cognatus* from each sampling station was moderately to highly diversified (H = 0.490 to 0.900, π = 0.0010–0.0034) except for ST1 and 14. Interestingly, the low level of genetic diversity in ST1 and 14 was apparent only in the matrilineal COI marker (H = 0.118 and 0.216; π = 0.0004 and 0.0008, respectively). A moderate level of genetic diversity was deemed for *U. cognatus* from each geographical region based on the respective genetic marker, indicated by the moderate-to-high H and relatively low-to-moderate π within the respective regions (Table 1). However, a much lower COI divergence of *U. cognatus* from ST1 (WCT) (H = 0.118, π = 0.0004) resulted in a reduced level of the overall COI genetic diversity within the WCT (H = 0.249, π = 0.0007). In general, the RAG1 sequences showed a higher H but lower π than the COI data set in most of the sampling stations.

### Phylogenetic relationship and population structure

**Figure 2:**
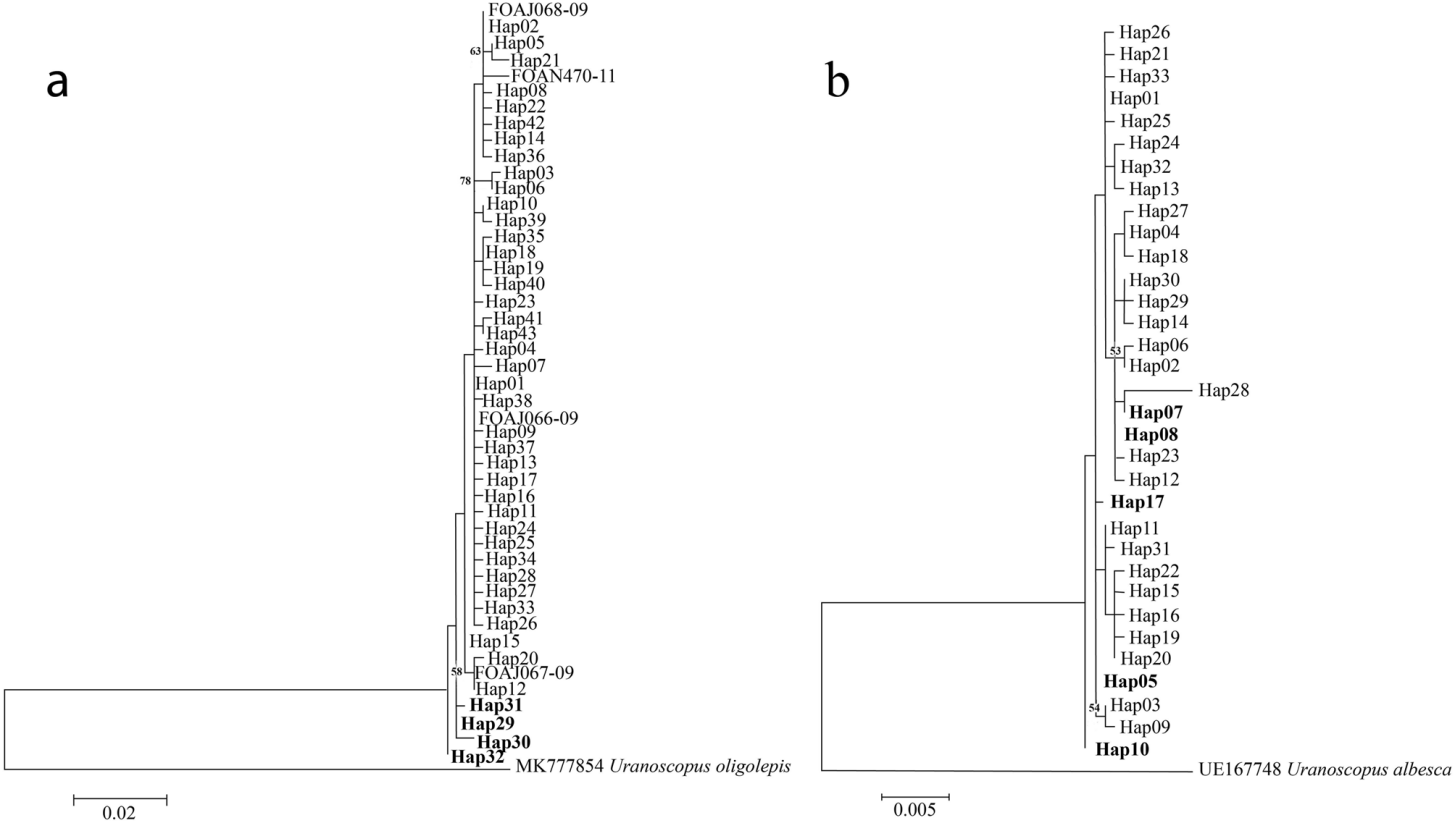
ML gene trees of *Uranoscopus cognatus* inferred from (**a**) CO1 (**b**) RAG1 sequences, constructed in MEGA 6.0 (https://www.megasoftware.net/resources). Branches were drawn to scale and bootstrap values < 50% were not shown. Haplotypes found in the west coast Thailand (WCT) were bold. FOAJ066-09, FOAJ067-09, FOAJ068-09 and FOAN470-11 were from Indonesia (BOLD sequences).

**Figure 3:**
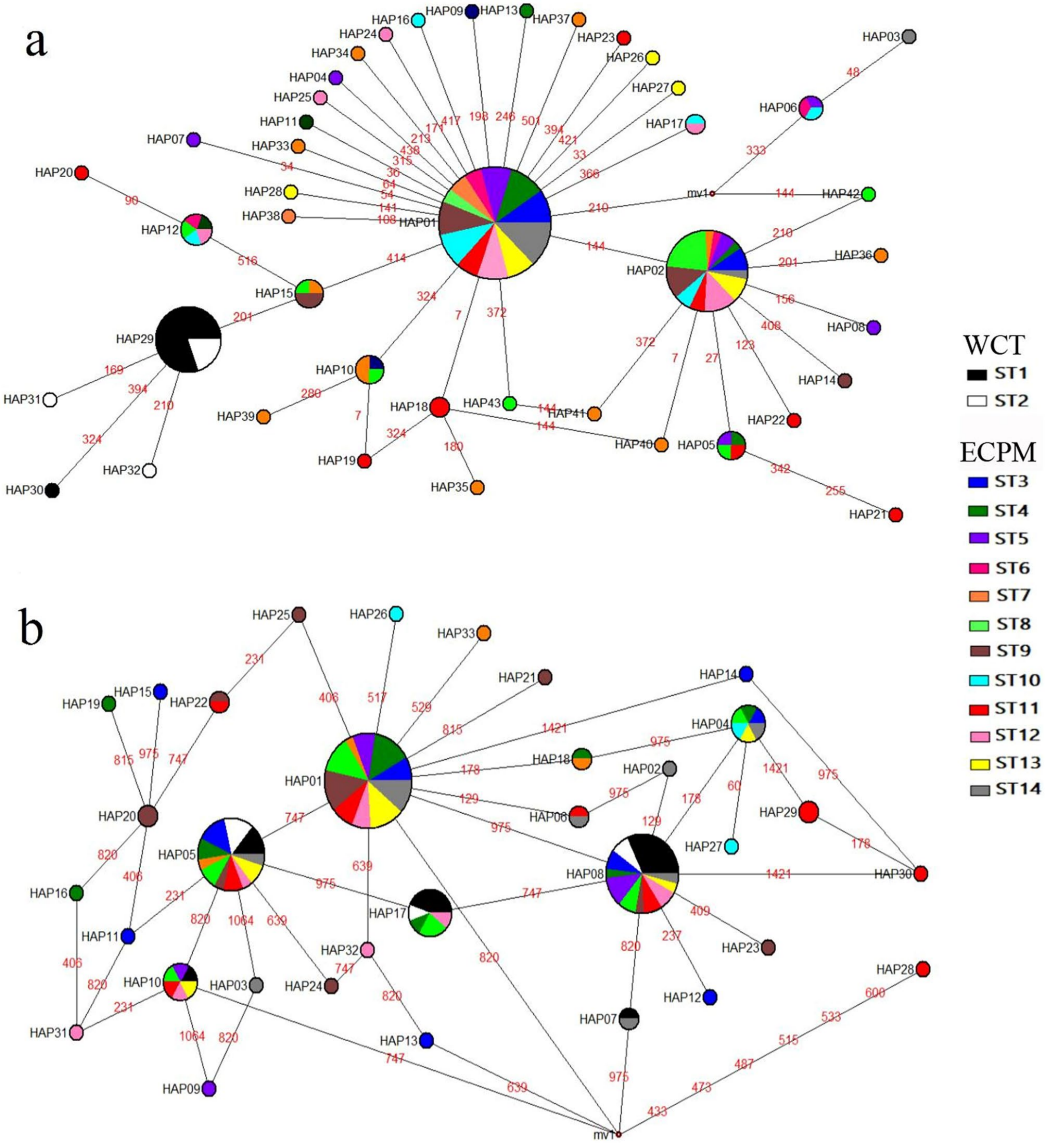
Haplotype network diagrams of *Uranoscopus cognatus* inferred from (**a**) CO1 (**b**) RAG1 sequences from the west coast Thailand (WCT) and the east coast Peninsular Malaysia (ECPM), constructed in Network v5.0.1.1 (https://www.fluxus-engineering.com/sharenet.htm). *Mv* median vector. Numbers in red are nucleotide mutation sites.

The ML COI gene tree revealed segregation of the WCT and ECPM haplotypes into separate clades with a relatively low bootstrap value (< 50%) (Fig. 2a); in contrast, the RAG1 gene tree showed ambiguous genetic partitioning, where the haplotypes from both regions were clustered into a single clade (Fig. 2b). Similarly, the four COI haplotypes that were private to the WCT (Hap29–32) were separated from haplotypes of ECPM and were placed as the external nodes in the MSN diagram (Fig. 3a), while genetic partitioning associated with the geographical region was not observed in the RAG1 haplotype network (Fig. 3b). The COI haplotypes of *U. cognatus* from Indonesia (INDO) were clustered closely with those from ECPM as they were either very close or totally identical (Fig. 2a).

SAMOVA based on the COI sequences revealed that the proportion of the total genetic differences between groups (*F*_CT_) was the highest (66.98%) when k = 2, separating the ECPM and WCT. In contrast, SAMOVA based on the RAG1 sequences revealed a much lower genetic subdivision between groups (*F*_CT_) (12.85–15.56%, for k = 2 to 5) and among populations (*F*_SC_) (-1.30–0.61%), yet a higher proportion of genetic variance within populations (*F*_ST_) (83.98–87.05%). The inter-regional pairwise comparisons Ф_ST_ (WCT *vs*. ECPM) based on the COI data set were all statistically significant, however, based on the nuclear DNA data set, five pairwise comparisons were statistically significant (involving only ST1) (Table 2). None of the intra-regional pairwise comparisons Ф_ST_ were statistically significant, except for ST8–ST14 based on the COI data set and ST10–ST13 based on the RAG1 data set. Genetic differentiation of *U. cognatus* from the WCT and ECPM was further evident based on H_ST_, N_ST_, K_ST_* (all estimates were significant at P < 0.05), and the relatively low gene flow (Nm) estimates inferred by both DNA markers (COI: H_ST_ = 0.142, N_ST_ = 0.741, K_ST_* = 0.220, Nm: 2.81 for haplotype-based statistics, 0.18 for sequence-based statistics; RAG1: H_ST_ = 0.055, N_ST_ = 0.177, K_ST_* = 0.047, Nm: 3.86 for haplotype-based statistics, 1.16 for sequence-based statistics).

**Table 2:** Population pairwise comparison Ф_ST_ among 14 sampling stations (ST) of *Uranoscopus cognatus* inferred from the partial CO1 (below diagonal) and RAG1 regions (above diagonal).

	ST	1	2	3	4	5	6	7	8	9	10	11	12	13	14
WCT	1		− 0.005	0.120	0.210*	0.174	N/A	0.345*	0.147	0.282*	0.198	0.130	0.177	0.275*	0.230*
	2	0.067		0.018	0.088	0.122	N/A	0.223	0.052	0.168	0.264	0.052	0.043	0.189	0.160
ECPM	3	0.838*	0.780*		− 0.038	− 0.034	N/A	− 0.019	− 0.042	− 0.003	0.157	− 0.028	− 0.049	− 0.025	− 0.013
	4	0.789*	0.719*	− 0.017		− 0.015	N/A	− 0.056	− 0.023	− 0.028	0.225	− 0.011	− 0.036	− 0.038	− 0.012
	5	0.744*	0.659*	− 0.022	− 0.01		N/A	0.011	− 0.043	0.032	0.213	− 0.033	− 0.046	− 0.042	− 0.051
	6	0.774*	0.648*	− 0.002	− 0.039	− 0.033		N/A	N/A	N/A	N/A	N/A	N/A	N/A	N/A
	7	0.641*	0.548*	− 0.017	0.005	0.001	− 0.014		0.011	− 0.009	0.265	− 0.039	− 0.013	− 0.055	− 0.031
	8	0.721*	0.631*	0.079	0.098	0.037	0.058	0.038		0.039	0.233	− 0.014	− 0.035	− 0.038	− 0.020
	9	0.792*	0.725*	− 0.011	0.001	− 0.014	− 0.006	− 0.001	0.02		0.295	0.024	0.007	0.009	0.029
	10	0.771*	0.693*	− 0.016	− 0.03	− 0.016	− 0.068	0.005	0.094	0.001		0.081	0.263	0.317*	0.213
	11	0.652*	0.552*	0.005	0.009	− 0.004	− 0.01	− 0.013	0.012	− 0.002	0.017		− 0.013	− 0.016	− 0.025
	12	0.771*	0.698*	− 0.026	− 0.021	− 0.019	− 0.03	− 0.002	0.044	− 0.029	− 0.027	− 0.001		− 0.033	− 0.004
	13	0.815*	0.746*	− 0.031	− 0.014	− 0.023	− 0.007	− 0.003	0.071	− 0.013	− 0.014	0.006	− 0.025		− 0.041
	14	0.854*	0.801*	0.005	− 0.01	0.003	− 0.028	0.023	0.171*	0.056	− 0.028	0.052	0.016	0.009	

*Significant at P < 0.05.

*WCT* west coast Thailand, *ECPM* east coast Peninsular Malaysia, *N/A* Data not available.

### Historical demography

All sampling stations recorded a negative value in either one or both neutrality tests of Tajima’s *D* and Fu’s *Fs* (several were statistically significant) except for ST2, based on the RAG1 sequences (Table 1). The Harpending’s raggedness index (Hri) and the sum of squared deviations (SSD) exhibited statistically non-significant values for all sampling stations and the overall data sets. Mismatch distribution analysis showed a unimodal pattern based on the respective molecular data set and geographical regions (Supplementary Fig. S1). The estimated tau (τ) values based on the COI sequences were 3.000 in the WCT and 0.998 in the ECPM, and based on the RAG1 sequences, they were 1.283 in the WCT and 1.773 in the ECPM. Following the equation of T = τ/2μk, the calculated time for population expansion based on the COI sequences was 158,700 and 272,717 years ago (ya) in the WCT and 52,794 and 90,723 ya in the ECPM, while based on the RAG1 dataset, the population expansion time was estimated to be around 463,773 ya in the WCT and 640,895 ya in the ECPM.

### Morphological analysis

**Figure 4:**
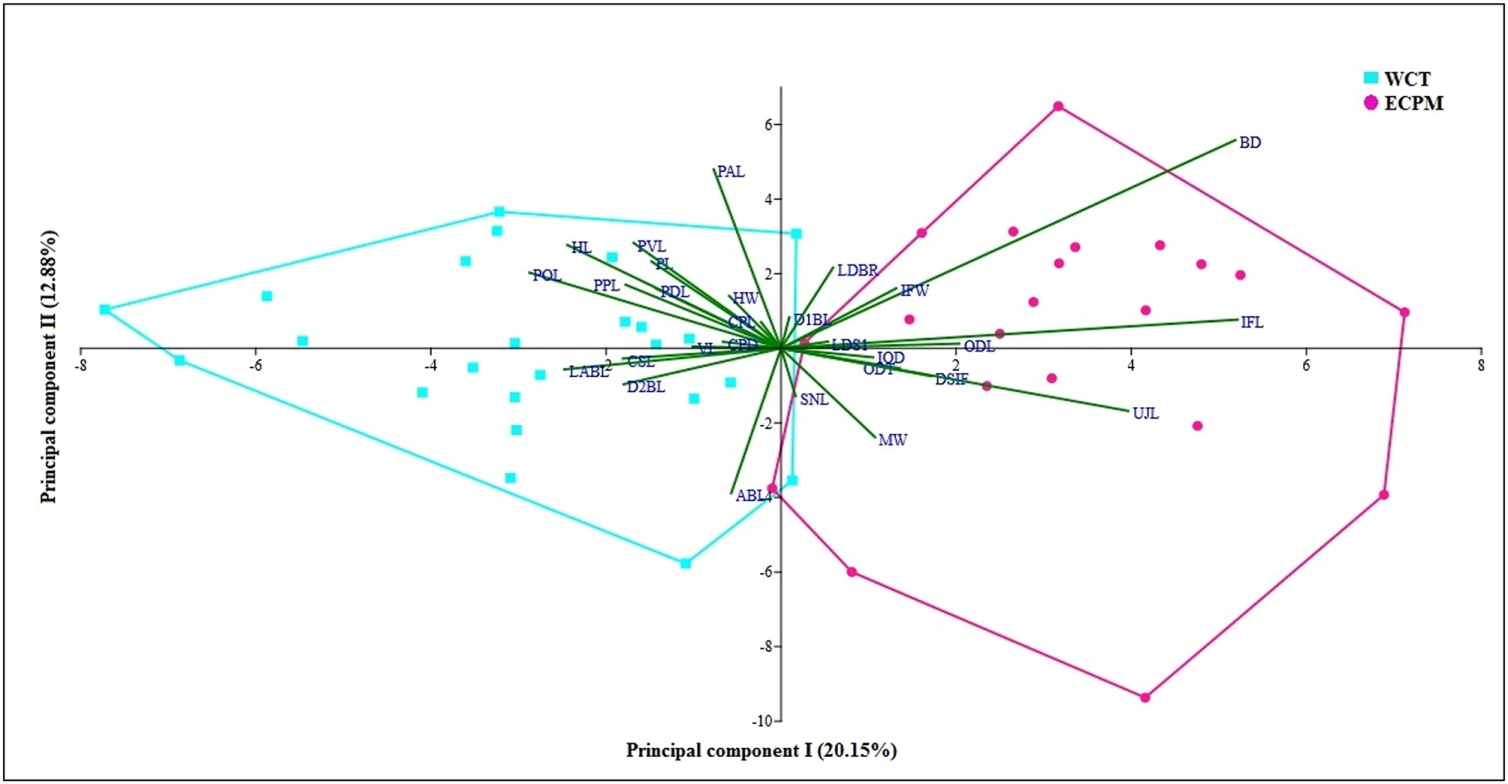
Principal component analysis depicting the first two axes of morphological variation of *Uranoscopus cognatus* from the west coast Thailand (WCT) (n = 24) and the east coast Peninsular Malaysia (ECPM) (n = 21), created in PAST v3.24 (http://priede.bf.lu.lv/ftp/pub/TIS/datu_analiize/PAST/2.17c/download.html).

Principal component analysis (PCA) based on 29 morphometric variables discriminated allopatric *U. cognatus* into distinct clusters at the principal component (PC) I (20.15% of total variance) (Fig. 4), whilst the rest of the PC showed certain degrees of overlap. The four variables with relatively high loadings on PCI were body depth (BD) (0.485), interorbital fossa length (IFL) (0.430), upper jaw length (UJL) (0.348), and postorbital length (POL) (-0.265); however, BD was excluded from our discussion as it is usually influenced by sex. The IFL and UJL were negatively correlated with POL, where an individual with a long IFL and UJL had a short POL, and vice versa. In this case, *U. cognatus* in the WCT was characterized with a short IFL and UJL but a long POL; in contrast, those from the ECPM had a long IFL and UJL but a short POL (Fig. 3, Supplementary Table S1).

The 12 meristic characteristics were indistinct for allopatric *U. cognatus*, except for the number of supracleithral spines, where the majority of *U. cognatus* from the ECPM (83%) and WCT (47.6%) had three and four supracleithral spines, respectively (Table 3). The selected characteristics are presented in Table 3. The number of embedded oblique scale rows on the body was 45–66 (WCT) and 44–64 (ECPM) (scales were only found on the body while the head, breast, and belly were naked). Cirri were short on the edges of the upper (poorly developed) and lower lips (6–18 (WCT) and 7–17 (ECPM)), and *U. cognatus* from both geographical regions had five pelvic fin rays, one subopercular spine and two basipterygial processes.

**Table 3:** Selected meristics characters of *Uranoscopus cognatus* from the west coast Thailand (WCT) and east coast Peninsular Malaysia (ECPM).

Meristics	Frequency			
**Number of supracleithral spines**	**3**	**4**	**5**	**6**
WCT	1	20	3	
ECPM	10	6	3	2
**Number of preopercular spines**	**3**	**4**	**5**	
WCT	1	20	3	
ECPM	2	18	1	
**Number of dorsal fin rays**	**iii, 13**	**iii, 14**	**iv, 13**	**iv, 14**
WCT	8	16		
ECPM	6	10	2	3
**Number of anal fin rays**	**12**	**13**	**14**	
WCT	1	21	2	
ECPM		18	3	
**Number of pectoral fin rays**	**15**	**16**	**17**	
WCT	3	19	2	
ECPM		19	2	
**Number of branched caudal rays (upper + lower)**	**6 + 6**	**6 + 7**	**7 + 7**	**8 + 7**
WCT	20		3	1
ECPM	16	1	2	2

## Discussion

An overall moderate level of genetic diversity was observed for *U. cognatus* from the WCT and ECPM, considering the moderate-to-high H and low-to-moderate π (Table 1), except for ST1 and 14. A low level of genetic variation in these populations was apparent but only in the maternally inherited COI gene marker, possibly due to stochastic factors or anthropogenic stressors. Most of the *U. cognatus* populations assessed in this study were deemed as still healthy and abundant; however, the low level of COI genetic diversity observed in ST1 and 14 should warrant special attention from the authorities to ensure a sustainable bioresource in the long term. Conservation measures are critical because the adult stargazer is strongly site-associated and has a limited capacity for dispersal and mobility, thus, it is vulnerable to site-specific benthic impacts such as trawling and dredging^4^. Nevertheless, low genetic diversity along the Andaman Sea has been recorded in commercially important species and is generally attributed to overharvesting, leading to population bottleneck and a subsequent genetic reduction in the total gene pool^28,34,36^. Ignoring such ecological or fishing pressures will likely cause adverse effects to marine resources, for instance, depletion of marine species along the WCT^56–58^. A recent study recorded severe population size reduction in *U. cognatus* from the central-eastern coast of India^4^ due to overfishing and unsustainable trawling activities. Consequently, conservation measures must be taken because higher fishing pressure is likely to further deteriorate diversity levels and population size in the future^5,59,60^.

A moderate-to-high H and low-to-moderate π observed in the present study suggests a recent population expansion from a past bottleneck event^30,31,61^. This finding is further corroborated by the negative values in the Tajima’s *D* and/or Fu’s *Fs* tests (except in ST2) (Table 1), with a unimodal pattern of the mismatch distribution (Supplementary Fig. S1), statistically non-significant Harpending’s raggedness index and sum of squares deviation, and a star-like pattern of the minimum spanning network (Fig. 3). Historical events during the Pleistocene epoch (2,580,000 to 11,700 ya) could have shaped the genetic distribution of the *U. cognatus* populations observed in the present study. Our demographic analyses revealed two bouts of population expansion: an older expansion uncovered by the nuclear DNA and a more recent one uncovered by the mitochondrial DNA markers. The nuclear data revealed a population expansion dated back to 463,777 and 640,895 ya while the mitochondrial DNA estimated expansion dating to 52,000 and 272,000 ya. The different temporal scale estimated from mitochondrial and nuclear DNA was not unexpected due to differences in their effective population sizes and substitution rates^41^. The more rapid substitution rate in the COI marker obscured signals of older expansions for *U. cognatus* from both geographical regions, which were only revealed by nuclear DNA. Our finding was in concordance with the study on reef fishes inhabiting the Caribbean region and inferred from both mitochondrial and nuclear DNA^41^.

Understanding the life history of a species and its associated contemporary and paleo-environmental factors, as well as characteristics of the genetic markers employed in a study are particularly important in elucidating phylogenetic relationships and genetic distribution. Our molecular results indicated the close kinship of *U. cognatus* spanning the IWP, suggesting a common source of origin or ancestor, yet prolonged habitat isolation, resulting in a significant genetic divergence as detected by a more rapidly mutated genetic marker than the slower one. In particular, the presence of a significant matrilineal structure was found between the WCT and ECPM, and this partially agreed with the nuclear DNA marker. The sharing of nuclear DNA haplotypes in the allopatric populations and an evenly distributed shared polymorphism within the ECPM suggest the retention of ancestral polymorphism because of incomplete lineage sorting^62^. The nuclear DNA RAG1 gene is known to evolve slower^41,63^ than the COI gene, and needs a longer generation time to be fully fixed regionally. Given the massive effective population size of *U. cognatus* within IWP, even prolonged vicariance for tens of thousands of years can yield Ф_ST_ values lower than 0.5^25^, as seen in the present study based on RAG1 sequences (Table 2).

Mitonuclear disparity is often interpreted as a sex-biased dispersal of female philopatry and male-mediated gene flow^64,65^. However, we argue that this is not the case for *U. cognatus*. Like many other benthic species, stargazers live a bipartite life-cycle that begins with a pelagic larval phase and later a site-attached sedentary adult phase^4,9^. The adult stargazer is characterized by having an inactive benthic lifestyle^15,65,66^ and spends a greater part of its life in the sand or mud with limited mobility^4,15^. In addition, stargazers do not possess a visible swimming bladder after the adults’ flexion stage, leading to a sedentary lifestyle^15,65,66^ as described for the Atlantic stargazer (*U. scaber*) and the northern stargazer (*Astroscopus guttatus*). However, the life cycle and reproductive characteristics of *U. cognatus* are still poorly known. Although the sex-related dispersal pattern among aquatic organisms has been documented in elasmobranch, horseshoe crab, eels, sea-snake and turtle^67–71^, it has never been reported in stargazer. Thus, more studies on uranoscopids should be conducted to uncover more information on this benthic creature.

Despite the absence of physical barriers to dispersal in the marine realm, our study showed that the populations of *U. cognatus* were bounded by a genetic barrier in the central division (central part of the IWP region) of the Indonesian Archipelago, known as the Indo-Pacific Barrier (IPB), through concomitant changes in oceanographic current, presumably prohibiting gene flow to happen between regions^21^. This is in concordance with the genetic break of the Indian-Pacific ocean-basin hypothesis^21^, and in accordance with earlier observations of several marine species including reef fish^29–32^, invertebrates^33–35^, seahorses^36^ and pelagic fish^37^. Nevertheless, several findings on fish that showed low genetic differentiation within the IWP region were also reported and associated with the highly dispersive life history stages that contribute to high rates of gene flow among populations, for instance in moray eels^26^, Indian mackerel (*Rastrelliger kanagurta*)^27^, and Japanese scad (*Decapterus maruadsi*)^28^. To better comprehend the evolutionary history of *U. cognatus* and the level of structuration at the species level, collecting more samples from outside of the WCT and ECPM is therefore highly required.

Interestingly, the genetic proximity of *U. cognatus* from the ECPM with those from central and west Java, Indonesia was apparent (Fig. 2a), suggesting a common source of origin or a long-range dispersal ability of planktonic larvae about 2,500 km apart. Long-range dispersal is possible via the seasonally reversing monsoon-generated currents through the Flores and the Java Sea into the South China Sea^72^, permitting a repeated mixing and homogenizing of the gene pool of *U. cognatus* between these regions. Genetic propinquity of the marine species inhabiting the South China Sea and the Java Sea was also reported in mantis shrimp (*Haptosquilla pulchella*)^73^, the anemonefish (*Amphiprion ocellaris*)^31,74^, and the Pearse’s mudskipper (*Periophthalmus novemradiatus*)^75^.

Fishes are vulnerable to environmental distortion but able to adapt to changing surroundings through modification of their morphology, physiology and behavior. Phenotypic plasticity may, in some instances, reveal a high pliancy in morphological characteristics in response to a different environmental condition, such as availability of prey and water temperature^76,77^. The present study uncovered the phenotypic divergence of *U. cognatus* from the WCT and ECPM, characterized mainly by the interorbital fossa length (IFL), upper jaw length (UJL), postorbital length (POL), and the number of supracleithral spines. The IFL and UJL are among the variables with the highest loadings on the PCI, suggesting evolution in the premaxillary protrusion, likely associated with the mechanism for upper jaw movement in relation to biomechanical approaches to feeding (suction feeding). However, the rest of the morphometric and meristic characteristics were indistinct for *U. cognatus* from the WCT and ECPM, suggesting a close kinship or a shared common ancestor between the individuals, but with some phenotypic modifications for a better adaptation to the benthic lifestyle^15^. A low morphological divergence was also reported in the Atlantic stargazer (*U. scaber*) collected from five different seas around the Mediterranean Sea basin^78^ and in *U. marmoratus* populations within Palk Bay India^53^, suggesting specific local adaptations to environmental changes.

## Conclusion

The genetic and morphological data of *U. cognatus* in this study provide strong evidence for a shared common origin or ancestor of *U. cognatus* within the IWP and genetic breakdown by the IPB for a long period has likely subdivided the populations into distinct WCT and ECPM lineages, followed by subsequent range expansion within respective regions. The retention of ancestral polymorphism within the IWP was likely due to incomplete lineage sorting in allopatric populations. The moderate genetic diversity estimated for each region, in addition to past population expansion events, indicated that *U. cognatus* within the scope of this study was still healthy and abundant, except for ST1 and 14. Although a low genetic diversity was apparent only in the matrilineal COI marker, special attention from the authorities is required to prevent further depletion of bioresources. Heterogeneous biotic and/ or abiotic conditions of the WCT and ECPM might have influenced *U. cognatus* differently, as apparent by morphological modifications to better adapt and survive in the benthic ecosystem. The current results are important for habitat and fisheries resource management, highlighting the effectiveness of incorporating highly conserved *vs.* rapidly evolving molecular markers, and morphological characteristics in elucidating the population genetics of benthic species within the IWP.

## Methods

### Sample collection and preservation for molecular study

Tissue samples were obtained from dead/preserved specimens, and no experiments on live animals were performed in this study. Random samples of *U. cognatus* were collected from the west coast of Thailand (WCT) and east coast of Peninsular Malaysia (ECPM) (Fig. 1b, Supplementary Table S2). Samples from the WCT consisted of two localities (Sampling station (ST) 1–2), while samples from the ECPM consisted of 12 localities (ST3-14). Five random samples of *U. cognatus* from the ECPM were deposited at the South China Sea Repository and Reference Centre (RRC), Institute of Oceanography and Environment (INOS), Universiti Malaysia Terengganu (UMT) with voucher specimen reference numbers UMTGen1318 to UMTGen1323. A small portion of the pectoral fin was cut and cleaned before being preserved in 1.5 mL microcentrifuge tubes containing 95% ethanol solution at room temperature until further analysis.

### Genomic DNA extraction and polymerase chain reaction (PCR) optimization

Total genomic DNA was isolated from fin tissues by a standard salting-out protocol^79^ and amplified at the partial mitochondrial DNA cytochrome c oxidase subunit I (COI) gene using the primers FishF2 and FishR2^80^ and the single-copy nuclear recombination activating gene 1 (RAG1) using the primers RAG1F1 and RAG1R2^81^. The PCR amplification for both DNA markers was carried out in a final volume of 25 µL consisting of 2.0 µl genomic DNA (50 ng/µL), 0.5 µM of each primer, 9.5 µl sterile ultrapure nano water (ddH_2_O) and 12.5 µl MyTaq DNA Polymerase (Bioline, Meridian Bioscience Inc., United Kingdom). The PCR thermal regime for the CO1 consisted of an initial incubation at 95 °C for 2 min (min), followed by 35 cycles of 94 °C denaturation for 30 s (sec), 54 °C annealing for 30 s, and 72 °C extension period for 60 s, followed by a 70 °C final extension for 10 min before termination at 4 °C^80^. The PCR thermal protocol for RAG1 consisted of an initial incubation at 95 °C for 3 min, followed by 40 cycles of 94 °C denaturation for 30 s, 60 °C annealing for 45 s, and 72 °C extension period for 90 s, followed by a final extension at 72 °C for 10 min, and a final hold at 4 °C (modified from Mat Jaafar et al.^82^). PCR products were visualized on a 1.7% agarose gel stained with 3 µL SYBr safe and were sequenced using the forward primer for the COI and both the forward and reverse primers for RAG1 by the service provider, Apical Scientific Laboratories Sdn. Bhd. Selangor, Malaysia.

### Molecular data analysis

#### Sequence alignment and validations

Multiple sequences were aligned and trimmed using ClustalW implemented in MEGA 6.0^83^. A total of 231 and 171 individuals were successfully amplified for the COI (534 base pairs (bp)) and RAG1 (1,426 bp) genes, respectively. To ensure that the sequences were aligned correctly, they were first translated into a protein sequence (no stop codon or indel was found). Then, the sequence identity was verified through the BOLD Identification System. To examine the degree of heterogeneity among the data sets and to validate whether the two datasets could be combined for analysis, the incongruence length difference for each data set was tested with 1,000 replications implemented in the partition homogeneity test^84^ using heuristic searches in PAUP* v4.0b10^85^. The partition homogeneity test returned a significant incongruence in the phylogenetic signal among the CO1 and RAG1 sequences (P = 0.01), suggesting that the data sets should be analyzed separately. All haplotype sequences obtained in this study were deposited in the GenBank database with accession numbers MK728892 to 728934 (COI) and MN649790 to 649822 (RAG1).

#### Genetic diversity

The aligned sequences were screened for nucleotide variable sites, parsimony informative sites, the number of haplotypes (nh), and amino acid substitutions in DnaSP 5.10^86^. The genetic diversity indices, namely haplotype diversity (H) and nucleotide diversity (π), were calculated in Arlequin v3.5^87^.

#### Phylogenetic relationship and population structure

The best nucleotide substitution models with the lowest BIC (Bayesian Information Criterion) score for the COI and RAG1 data sets were the Hasegawa-Kishino-Yano + Gamma (HKY + G)^88^ and Kimura 2-parameter (K2P)^89^ models, respectively. The phylogenetic relationships of *U. cognatus* were assessed by constructing a maximum likelihood (ML) tree in MEGA 6.0. The robustness of the statistical support for the ML tree branch was determined by 1,000 bootstrap replicates^90^. To comprehend the phylogenetic relationship of *U. cognatus* from a wider geographical range, the available COI haplotypes from Indonesia (four sequences in the BOLD database: FOAJ066-09, FOAJ067-09, FOAJ068-09 and FOAN470-11) and Australia (two sequences: FMVIC082-07, and FOAO1380-18) were included, and *U. oligolepis* (MK777854) was used as an outgroup taxon. Several validation tests (BLAST on NCBI database, phylogenetic tree, and genetic distance) were conducted on the two sequences from Australia and showed that they were probably misidentification of *U. oligolepis*, thus, they were excluded from this study. Meanwhile, the only RAG1 GenBank sequence of *U. albesca* (EU167748) was included as an outgroup taxon in the RAG1 data set. The relationships of haplotypes were further elucidated based on a median-joining calculation using the Minimum Spanning Network (MSN) analysis implemented in Network version 5.0.1.1^91^.

To identify similar groups of populations and to evaluate the amount of genetic variation among the partitions, a spatial analysis of molecular variance was conducted in SAMOVA v1.0^92^. The partition scheme of groups (k) was determined based on the highest variances among groups (F_CT_), incorporating information on haplotype divergence and geographical proximity. The population pairwise comparison Ф_ST_ that calculates the genetic differentiation among sites was determined in Arlequin v3.5.2.2, and statistically significant pairwise comparisons were tested with 10,000 permutations to estimate the departure from the null hypothesis of genetic homogeneity. The significant probability values were adjusted by performing the False Discovery Rate Procedure (FDR) at α = 0.05, which controls the family-wise error rate (FWER), a conservative type I error rate originating from multiplicity^93^.

The genetic differentiation among populations was assessed by employing haplotype-based statistics, H_ST_^94^ and sequence-based statistics, N_ST_^95^ and K_ST_* and the significance levels were estimated using permutation tests with 1,000 replicates^96^ in DnaSP 5.10. The estimates of gene flow (Nm) based on both haplotype-based and sequence-based statistics were derived from the same program. The genetic distance estimates between the sampled stations were calculated in MEGA 6.0.

#### Historical demography

The historical demographic changes in the effective population sizes were assessed based on the neutrality tests Tajima’s *D*^97^ and Fu’s *Fs*^98^ statistics, mismatch distribution, Harpending’s^99^ raggedness index (Hri), and the sum of squared deviations (SSD) estimates. The neutrality tests of Tajima’s *D* and Fu’s *Fs* were used to evaluate the deviation from the neutral expectation that may arise from historical population range expansion or mutation-drift disequilibrium and were calculated in Arlequin v3.5.2.2. Mismatch distribution indicated whether *U. cognatus* was demographically stable, expanding, or decreasing over time^100–102^ and was estimated in DnaSP 5.10. The Hri and SSD were computed in Arlequin v3.5.2.2 to evaluate if the sequence data significantly diverged from the assumptions of a population expansion model. The historical demographic parameters of τ (relative time since population expansion) were computed in Arlequin v3.5.2.2. The τ value was used to estimate the actual time (T) since population expansion using T = τ/2μk, where μ is the mutation rate per site per generation and k is the sequence length^103^. In this study, two mutation rates were used for CO1 (*i.e.* 1.03% and 1.77% per million years), while a mutation rate of 0.097% per million years was used for RAG1^63^.

#### Morphological analysis

**Figure 5:**
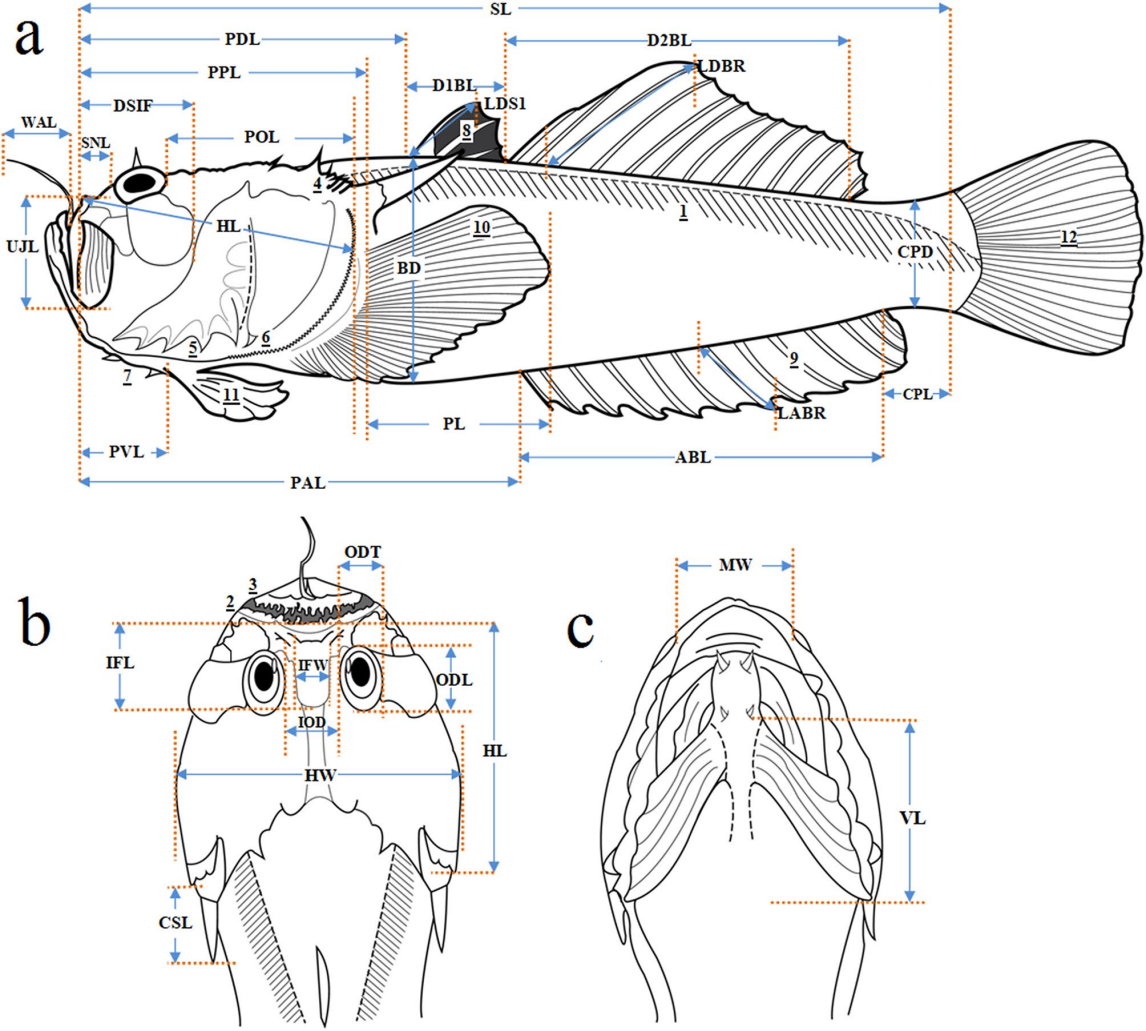
Linear morphometric measurement and meristic counts of *Uranoscopus cognatus* by (**a**) body side, (**b**) dorsal head view and (**c**) ventral head view. *SL* Standard length, *BD* body depth, *HL* head length, *HW* head width, *ODL* orbit diameter longitudinal line, *ODT* orbit diameter transversal line, *IOD* interorbital distance, *IFL* interorbital fossa length, *IFW* interorbital fossa width, *DSIF* the distance between the snout and the posterior margin of second infraorbital, *UJL* upper jaw length, *SNL* snout length, *MW* mouth width, *POL* postorbital length, *WAL* worm-like appendage length, *CSL* cleithral-spine length, *PL* pectoral fin length, *VL* pelvic fin length, *D1BL* first dorsal base length, *D2BL* second dorsal base length, *LDS1* length of first dorsal spine, *LDBR* length of longest dorsal branched ray, *ABL* anal base length, *LABR* length of longest anal branched ray, *PDL* predorsal length, *PAL* preanal length, *PPL* prepectoral length, *PVL* prepelvic length, *CPL* caudal peduncle length, *CPD* caudal peduncle depth; number of scale rows on body (1), upper lip fimbriae (2), lower lip fimbriae (3), supracleithral spines (4), preopercular spines (5), subopercular spines (6), basipterygial processes (7), dorsal fin rays (8), anal fin rays (9), pectoral fin rays (10), pelvic fin rays (11), and branched caudal rays (12).

Representative samples from the WCT (N = 24) and the ECPM (N = 21) were analyzed for phenotypic variation by using a total of 42 morphometric measurements, and meristic counts were taken on the left side of preserved specimens. Thirty linear measurements (Fig. 5), which represent the fish body dimensions, were taken using a digital caliper and the data was recorded to the nearest 0.01 mm and proportioned to the standard length (SL) or head length (HL). A total of 12 meristic counts were used (Fig. 5, Supplementary Table S3). The counting and measuring methods were adopted from Hubbs and Lagler^104^ and Kishimoto^105,106^, except for the head width, interorbital distance, interorbital fossa width and cleithral-spine length, where we followed Gomon and Johnson^107^, and the mouth (or gape) width was performed as in Rainboth^108^. The terminology of the morphological features was selected according to Pietsch^11^, Imamura and Matsumura^109^, and Vilasri^15^.

Principal component analysis (PCA) was carried out on the standardized data (*i.e.* the relative percentage to the standard or head length^53,110^) to equalize the variance among the groups, using the Paleontological Statistics Software Package (PAST) v3.24^111^. PCA analysis was carried out essentially by calculating the linear combinations of the original variables to create a ‘morphospace’ by plotting the specimens on the first few major axes of the morphological variations (PC axes), which represent the majority of the variability between specimens, while maintaining the relevant variation between the data points^112^.

## Supplementary Information

Supplementary Information.

## Data Availability

All haplotype sequences obtained in this study were deposited in the GenBank database with accession numbers MK728892 to 728934 (COI) and MN649790 to 649822 (RAG1). Other datasets generated during and/or analyzed during the current study are available from the corresponding author on reasonable request.
