# Supplementary material for: Genetic diversity, population structure and historical demography of the two-spined yellowtail stargazer (Uranoscopus cognatus)
Source: Sci Rep. 2021 Jun 25;11:13357. doi: 10.1038/s41598-021-92905-6 (PMC8233350; doi:10.1038/s41598-021-92905-6)
Supplement: Supplementary file 1 — Supplementary Information. [file 41598_2021_92905_MOESM1_ESM.pdf]

Genetic diversity, population structure and historical demography of the two-spined yellowtail stargazer (*Uranoscopus cognatus*)

Nur Ilham Syahadah Mohd Yusoff[1], Tun Nurul Aimi Mat Jaafar[1], Veera Vilasri[2], Siti Azizah Mohd Nor [3], Ying Giat Seah[1, 4], Ahasan Habib[1,5], Li Lian Wong[3], Muhd Danish-Daniel[3], Yeong Yik Sung[3], Abd. Ghaffar Mazlan[6], Rumeaida Mat Piah[1], Shahrol Idham Ismail[1], Min Pau Tan[1, 3]*

Supplementary Table S1: Morphometric measurements of *Uranoscopus cognatus* from west coast Thailand (WCT) and east coast Peninsular Malaysia (ECPM), expressed as percentages of standard length (SL) or head length (HL).

No	Morphometric character	WCT	ECPM
1	Standard Length (SL) (in mm)	71.86–147.54 (109.37, 24)	84.2–137.62 (113.15, 21)
		**Proportional to the SL (%) (mean, sample size)**	
2	Anal base length (ABL)	35.17–40.62 (38.12, 24)	33.67–43.63 (38.23, 21)
3	Body depth (BD)	20.58–28.09 (24.00, 24)	21.82–31.63 (26.99, 21)
4	Caudal peduncle depth (CPD)	11.07–12.67 (11.87, 24)	10.68–12.38 (11.37, 21)
5	Caudal peduncle length (CPL)	9.03–15.78 (11.75, 24)	8.84–13.25 (11.58, 21)
6	Cleithral-spine length (CSL)	8.66–13.86 (10.83, 24)	8.03–11.62 (9.86, 21)
7	First dorsal base length (D1BL)	8.45–19.03 (10.92, 24)	9.15–12.85 (11.11, 21)
8	Second dorsal base length (D2BL)	36.25–43.29 (40.29, 24)	36.10–43.26 (39.01, 21)
9	Head length (HL)	32.01–34.86 (33.42, 24)	29.86–34.46 (32.11, 21)
10	Head width (HW)	21.70–26.04 (23.55, 24)	21.99–26.54 (23.59, 21)
11	Length of longest anal branched ray (LABR)	9.33–14.34 (11.89, 24)	8.41–12.62 (10.46, 21)
12	Length of longest dorsal branched ray (LDBR)	16.20–20.51 (18.38, 24)	14.87–23.79 (18.78, 21)
13	Length of first dorsal spine (LDS1)	6.22–12.81 (9.62, 24)	7.37–12.51 (9.90, 21)
14	Preanal length (PAL)	50.91–58.59 (55.25, 24)	51.34–57.87 (55.07, 21)
15	Predorsal length (PDL)	36.25–42.39 (38.91, 24)	36.02–40.47 (37.98, 21)
16	Pectoral fin length (PL)	21.75–28.61 (25.55, 24)	22.56–28.15 (24.70, 21)
17	Postorbital length (POL)	23.52–26.58 (25.14, 24)	21.49–25.15 (23.38, 21)
18	Prepectoral length (PPL)	32.29–34.99 (33.49, 24)	30.28–34.11 (32.59, 21)
19	Prepelvic length (PVL)	19.95–26.25 (23.04, 24)	20.11–24.74 (22.33, 21)
20	Pelvic fin length (VL)	16.30–19.73 (18.01, 24)	14.98–19.26 (17.35, 21)
		**Proportional to the HL (%) (mean, sample size)**	
21	The distance between the snout and the posterior margin of second infraorbital (DSIF)	38.01–45.00 (41.16, 24)	37.81–44.94 (42.38, 21)
22	Interorbital fossa length (IFL)	21.60–30.43 (26.27, 24)	27.54–29.90 (29.10, 21)
23	Interorbital fossa width (IFW)	6.78–10.68 (8.73, 24)	7.04–12.57 (9.43, 21)
24	Interorbital distance (IOD)	15.67–18.15 (17.21, 24)	15.85–20.09 (17.68, 21)

25	Mouth width (MW)	45.25–50.98 (47.79, 24)	39.17–52.10 (48.19, 21)
26	Orbit diameter longitudinal line (ODL)	15.74–18.92 (17.33, 24)	17.29–20.85 (18.51, 21)
27	Orbit diameter transversal line (ODT)	13.43–18.05 (15.61, 24)	15.09–18.03 (16.80, 21)
28	Snout length (SNL)	11.55–15.10 (13.15, 24)	11.80–15.46 (13.15, 21)
29	Upper jaw length (UJL)	41.55–48.59 (45.37, 24)	42.88–50.43 (47.17, 21)
30	Worm-like appendage length (WAL)	10.07–56.38 (34.86,18)	13.82–65.13 (39.54,19)

Supplementary Table S2: Sampling stations (ST), geographical coordinates and sampling dates of *Uranoscopus cognatus* from the west coast of Thailand (WCT) and the east coast of Peninsular Malaysia (ECPM).

Region	ST	Latitude (North)	Longitude (East)	Sampling Date
WCT	1	7°48'24.8"	98°25'50.6"	27.10.2016
	2	10°03'33.1"	98°23'14.5"	26.5.2017
ECPM	3	7°13'39.6"	102°54'12.0"	14.5.2016
	4	7°04'16.2"	102°50'10.2"	15.5.2016
	5	6°46'15.0"	103°29'30.0"	12.5.2016
	6	5°15'02.9"	103°17'07.0"	14.10.2018
	7	4°42'39.6"	104°22'17.4"	15.6.2016
	8	4°32'12.0"	104°20'57.6"	16.6.2016
	9	4°06'33.0"	104°17'34.2"	16.6.2016
	10	3°42'00.6"	104°38'22.8"	22.6.2016
	11	3°21'18.0"	104°27'50.4"	25.6.2016
	12	3°07'26.4"	104°48'04.8"	23.6.2016
	13	2°43'34.8"	104°32'34.2"	24.6.2016
	14	1°54'05.4"	104°55'14.4"	30.6.2016

Supplementary Table S3: Morphometric measurements and meristic counts of *Uranoscopus cognatus*

No.	Characters	Description
Morphometric measurements		
1	Standard Length (SL)	The length from the median anterior margin of the upper lip (disregarding the cirri) to the middle end of the hypural plate.
2	Body depth (BD)	The depth of body on perpendicular and measure from the origin of the first dorsal-fin base.
3	Head length (HL)	The length from the median anterior margin of the upper lip to the posterior end of the opercular membrane.
4	Head width (HW)	The width of opercular bone at middle position.
5	Mouth width (MW)	The width between the both of mouth angle side (closed mouth).
6	Interorbital fossa length (IFL)	The length of median point at the base of the upper lip to the posterior edge of the interorbital fossa.
7	Interorbital fossa width (IFW)	The width of the interorbital fossa and measure by passing the center of pupil on perpendicular.
8	Interorbital distance (IOD)	The distance between both side of upper edge of eyelid and measure by passing the center of pupil on perpendicular.
9	Orbit diameter longitudinal line (ODL)	The diameter of the longest orbit along the length from the anterior and posterior edges of eyelid edge.
10	Orbit diameter transversal line (ODT)	The diameter of the widest orbit from the upper and lower edges of eyelid edge.
11	Snout length (SNL)	The distance from the median anterior point of the upper lip to the anteriormost edge of the eyelid.
12	Upper jaw length (UJL)	The maximum length of the median anterior margin of the upper lip to the end posteriormost edge of the maxilla (the longest part in transverse) (the longest section along the oblique line).
13	Worm-like appendage length (WAL)	The length of a peculiar dermal appendage present at the central tip of the respiratory valve inside lower jaw by taken from extremity and middle base of appendage.
14	Cleithral-spine length (CSL)	The length of cleithral spine by measure from the tip of cleithral spine to the joint of opercular membrane.
15	Snout to posterior margin of second infraorbital (DSIF)	The distance between the snout and the posterior margin of second infraorbital
16	Postorbital length (POL)	The distance between the posteriormost edge of eyelid and the posterior end of opercular membrane.
17	First dorsal base length (D1BL)	

18	Second dorsal base length(D2BL)	The length of first and second of dorsal-fin bases, separating measurement for both parts. The first base is defined from the anterior surface of the first-spine base of first dorsal fin to the base of the preceding soft ray of second dorsal fin, whereas the second continues from the latter position to the base of the posteriormost ray.
19	Pelvic fin length (VL)	The length is measured from anteriormost pelvic base to the tip of the longest pelvic-fin ray.
20	Pectoral fin length (PL)	The length is taken from by the uppermost of pectoral base to the tip of the longest rays.
21	Anal base length (ABL)	Distance from origin of anal base to the base of the posteriormost ray.
22	Predorsal length (PDL)	The distance from the median anterior margin of the upper lip to the origin of the dorsal base of first dorsal base fin.
23	Preanal length (PAL)	The distance from the median anterior margin of the upper lip to the origin of the anal base.
24	Prepectoral length (PPL)	The distance from the median anterior margin of the upper lip to the base of the uppermost pectoral-fin ray.
25	Prepelvic length (PVL)	The distance from the median anterior margin of the upper lip to the base of the anteriormost pelvic-fin ray.
26	Length of longest anal branched ray (LABR)	The length from base to the tip of longest anal branched ray.
27	Length of longest dorsal branched ray (LDBR)	The length from base to the tip of longest dorsal branched ray.
28	Length of first dorsal spine (LDS1)	The distance from base to the tip of first dorsal spine ray.
29	Caudal peduncle depth (CPD)	The least depth of the peduncle.
30	Caudal peduncle length (CPL)	The distance from the middle end of anal base fin to the end of hypural plate.
Meristic counts		
1	Scale rows	Counted oblique scale rows from the anterior most row to that of the middle end of hypural plate (Left body side).
2	Upper lip fimbriae	

3	Lower lip fimbriae	Counted fimbriae of upper and lower lips, including rudimental elements.
4	Supracleithral spine	Number of prominent spine exposed from bone.
5	Preopercular spine	
6	Subopercular spine	
7	Basipterygial processes	
8	Dorsal-fin ray	Counted separately part of each spine and soft rays (Spines – unpaired and unsegmented elements, labeling design by roman numerals; soft rays – paired and segmented elements, labeling design by Arabic numerals). *For the last pterygiophore, supporting one or two ray elements): counted as 1 ½ if some aspects have 2 rays.
9	Anal-fin ray	Counted in a procedure similar to the dorsal-fin ray count.
10	Pectoral-fin ray	Due to pectoral and pelvic fin is double fin, counting all soft rays only the left fin of fish body.
11	Pelvic-fin ray	
12	Branched (upper + lower) at caudal fin ray	Counted the branched caudal rays by separate upper and lower portions (upper caudal rays - rays connected to upper hypural plate, epurals and neural spine; lower caudal rays - rays connected to lower hypural plate parhypural and hemal spine).

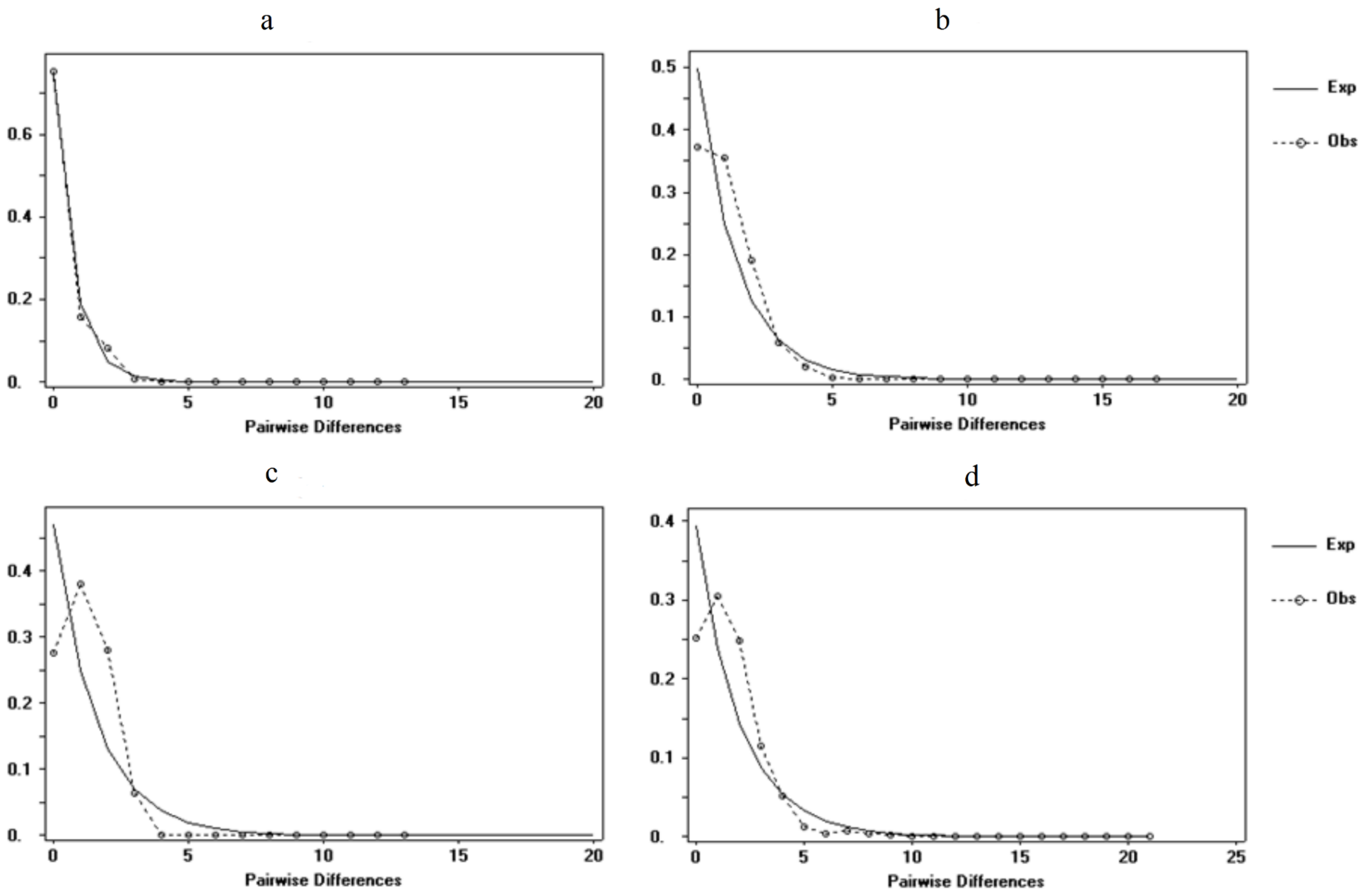

Supplementary Figure S1: Mismatch distribution (pairwise number of differences) of *Uranoscopus cognatus* based on the CO1 gene in (a) WCT and (b) ECPM; and RAG1 gene in (c) WCT and (d) ECPM, showing the expected observed pairwise differences between the sequences with the respective frequency. The graphs were created in DnaSP 5.10 (http://www.ub.edu/dnasp/).
